# From dust till dawn: patterns, motives, and risks of using smokable synthetic cathinones

**DOI:** 10.1186/s12954-026-01428-8

**Published:** 2026-02-25

**Authors:** Antonia Bendau, Paale Bournot, Felix Betzler, Christopher Clay, Jonas Desaga, Twyla Michnevich

**Affiliations:** 1 Department of Psychiatry and Neurosciences CCM, Charité – Universitätsmedizin Berlin, Corporate Member of Freie Universität Berlin and Humboldt Universität zu Berlin, Charitéplatz 1, 10117 Berlin, Germany; 2 sidekicks.berlin / Schwulenberatung Berlin gGmbH, Berlin, Germany; 3 Bundesinitiative Sexualisierter Substanzkonsum (BISS): Netzwerk für Prävention, Beratung, Erforschung und Behandlung e.V., Munich, Germany

**Keywords:** Monkey dust, Flakka, Flex, New psychoactive substances, Research chemicals, MDPV, Pyrovalerone, MDPHP, α-PHP, α-PHiP

## Abstract

**Background:**

Synthetic cathinones that are primarily smoked—such as pyrovalerone-type compounds, including MDPV, MDPHP, α-PHP, and α-PHiP, often referred to as “Monkey Dust”—have raised growing concern in clinical and harm reduction contexts due to their association with severe adverse psychological and behavioral effects. To date, detailed empirical user-level data on patterns of use and related aspects are absent. To address this gap, the present study aims to integrate first-hand perspectives and key characteristics of individuals using smokable synthetic cathinones to examine patterns of use and experiences associated with these substances.

**Methods:**

As part of a large cross-sectional online survey on synthetic cathinone use in general, this study investigated the use of smokable synthetic cathinones in Germany (March–May 2025). A sample of 107 participants who reported use within the past 12 months was analyzed in detail. Quantitative measures—combined with content analysis of open-text responses—captured sociodemographics, patterns, settings, and motives of use, mental and physical health, adverse effects, reduction efforts, and support needs.

**Results:**

The majority of individuals who used smokable synthetic cathinones matched typical chemsex profiles (male, homosexual, urban, highly educated), yet non-chemsex use populations were also identified. One quarter reported using at least once a week and had high rates of problematic or dependent use indicators. One third reported a current mental disorder and symptoms of depression and anxiety were common. Frequently reported adverse effects included psychotic symptoms, anxiety, and panic attacks—consistent with the clinical profiles (e.g., sympathomimetic characteristics) typical of smokable synthetic cathinones—and were particularly prevalent among those reporting frequent use. Around one third reported applying safer use strategies, and nearly half had initiated reduction or cessation efforts.

**Conclusions:**

This study provides the first systematic user-centered insights into the use of smokable synthetic cathinones, revealing heterogeneous populations with varying use patterns and risks. The findings highlight the need for targeted prevention and support strategies that address both chemsex-related and other emerging use profiles and settings.

**Clinical trial registration:**

The study was prospectively preregistered with the German Clinical Trials Register (DRKS; drks.de/search/en/trial/DRKS00035946) in February 2025.

**Supplementary Information:**

The online version contains supplementary material available at 10.1186/s12954-026-01428-8.

## Introduction

Emergency services in Germany (particularly in Berlin and Göttingen) have reported rapidly increasing numbers of severe incidents associated with substances sold under the name “Monkey Dust” [1, 2]. These presentations often involve extreme agitation, psychosis, and aggressive behavior. Locally rising trends have also been reported in the UK [3] and Finland [4]. This growing visibility is further mirrored by a pronounced increase in Google search interest for “Monkey Dust” in Germany since 2023 (see Supplementary Figure S1), suggesting heightened public attention and information-seeking related to these substances [5]. Despite this growing presence, there is a striking absence of scientific data regarding the patterns, motives, and risks associated with use of these substances.

Monkey Dust belongs to the chemical group of *synthetic cathinones* (synthetically produced amphetamine derivatives structurally related to cathinone, the active compound in the khat plant), which are colloquially referred to as "bath salts" and represent a large group of *new psychoactive substances* (NPS) [6–8]. A rough distinction can be made between synthetic cathinones that are *typically consumed nasally* (i.e., snorted)—such as “mephedrone” (4-MMC), 3-MMC, 2-MMC, 4-CMC, and 3-CMC—and those that are *usually smoked*. The latter include *methylenedioxypyrovalerone* (MDPV) and *3',4'-methylenedioxy-α-pyrrolidinohexiophenone* (MDPHP), commonly sold under the street names Monkey Dust, Cloud 9, Super Coke, PV/peeve, or Flex, differing based on region [1, 8, 9]. Several other smokable synthetic cathinones such as *alpha-pyrrolidinopentiophenone* (α-PVP), *alpha-pyrrolidinohexanophenone* (α-PHP), and *alpha-pyrrolidinoisohexanophenone* (α-PiHP) are further known as Flakka, Gravel, Zombie Drug, Alpha/Alfa, or Bloom [1, 8–10], For simplicity, all of these usually smoked synthetic cathinones with similar effect profiles will be referred to as *pyrovalerones* in the following, while noting that the broader cluster of smokable synthetic cathinones also includes other substances (e.g., *N-ethylpentedrone* (NEP)).^1^ The pyrrolidine ring of pyrovalerones enables smoking in freebase form (which is less effective for typically nasally used synthetic cathinones) and increases lipophilicity, facilitating blood–brain barrier penetration and enhancing psychoactive effects [13, 14]. They can also be administered intravenously, orally, and – less suitably due to hydrophobic properties – nasally or rectally.

There is a large and growing number of synthetic cathinones that are smokable due to their physicochemical properties, including numerous pyrovalerone derivatives (e.g., MDPV, MDPHP, MPHP, α-PVP, α-PHP, α-PHiP, 4-MeO-α-PVP, 4-F-α-PVP, PV8, α-PBP, and many related analogues) [13, 15]. These various smokable synthetic cathinones constitute a heterogeneous group with differing effects and associated risks. In the present study, they were nevertheless clustered as “smokable” or “usually smoked synthetic cathinones” to reflect naturalistic patterns of use, and enhance analytic utility, as individuals tend to report use at the level of perceived substance groups and routes of administration rather than specific chemical entities [16], and substance-specific stratification would have resulted in highly fragmented and variable, thus less interpretable, data.

Smokable synthetic cathinones commonly produce stimulant effects, with many compounds showing high potency [12, 14]. Reliable data on their dosage, duration, and effects are scarce (further complicated by the breadth of substances and the rapid emergence of new compounds, which constitutes an additional source of uncertainty), with most available evidence stemming from case reports, (post-mortem) toxicological analyses, and animal studies [7, 8, 17]. Desired effects seem to include increased energy, euphoria, sociability, disinhibition, and increased libido, as well as suppression of hunger and fatigue. Pharmacologically, pyrovalerones are strong dopamine and norepinephrine reuptake inhibitors, with, for example, MDPV having a potency 50–100 times higher than cocaine [10, 18]. These properties contribute to its intense euphoric effects and the high addiction potential. The rapid onset of effect, particularly via smoking/inhalation, and the intense but short-lived high assumedly contribute to compulsive use patterns and strong re-dosing tendencies [19].

Available evidence for some smokable synthetic cathinones describes somatic adverse effects including elevated heart rate, hypertension, hyperthermia, nausea, and tremor, as well as severe complications such as rhabdomyolysis, acidosis, renal failure, and cardiac arrest [9, 20, 21]. Acute intoxications seem to be often associated with psychomotor agitation, compulsive and bizarre behavior, and heightened aggression [21–25]. Psychotic symptoms—including hallucinations, paranoia, and delusions—are common and may persist for several days, sometimes after a single use [24]. After-effects include insomnia, depressive mood, irritability, and anecdotal reports of suicidality [12]. Sleep deprivation, compounded by repeated dosing and extended use sessions, may exacerbate these effects [26]. Elevated suicide rates have been observed in forensic analyses of deaths involving pyrovalerones [4]. Data on long-term effects are scarce, though anecdotal accounts suggest severe physical and mental deterioration in individuals with chronic use [2, 12].

The pharmacological and psychological risks are probably further compounded by the social and structural conditions under which pyrovalerones are consumed: In cities like Göttingen (Germany) and Stoke-on-Trent (UK), the substances circulate primarily among highly marginalized groups, including homeless individuals and those with limited access to healthcare or social support [2, 3]. In these contexts, pyrovalerones may sometimes replace more established substances such as heroin or crack cocaine [2, 3]. Instead of substitution based on pharmacological similarity, this may be driven by functional and situational considerations, including lower cost, high availability (partly attributable to the legal status of pyrovalerones in several countries), market dynamics, patterns of polysubstance use, and short-acting, potent stimulant effects of pyrovalerones [13, 21].

In parallel, emerging data from Berlin suggests growing popularity in the context of sexualized drug use ("chemsex"), particularly among men who have sex with men (MSM) [1]. Here, the substances’ strong stimulant and disinhibiting effects are likely sought to intensify and enhance sexual activities, contributing to extended sessions of drug-facilitated sex, often lasting several hours to days [27].

These divergent patterns underscore the need for differentiated prevention and intervention strategies tailored to distinct use groups and environments. This is currently obscured by the lack of empirical data. Existing knowledge is derived largely from media accounts, anecdotal reports, and isolated toxicological findings, leaving significant gaps in understanding patterns of use, contextual drivers, and perceived negative effects and harm [1, 28]. This study seeks to address this empirical gap by providing the first detailed analysis of smokable synthetic cathinone use, investigating use patterns, sociodemographic profiles, settings, underlying motives, experienced adverse effects, reduction efforts, and support needs associated with these substances. By centering first-hand perspectives of individuals who use them, the study aims to contribute to a more nuanced understanding of this complex topic.

## Methods

### Study design

Data were collected as part of a large cross-sectional observational study (*COMPASS—Consumption Motives, Patterns and Harm-Reduction Strategies Related to Synthetic Cathinones*) conducted between March 24 and May 24, 2025. Assessments were completed fully anonymously online using the SoSci-Survey platform [29]. The study focused on synthetic cathinones (with other substances considered only in relation to synthetic cathinone use) and included individuals who used synthetic cathinones as well as members of their social environments (e.g., people who interact with them or frequent settings where use occurs; own lifetime or current use was therefore not an inclusion criteria). Eligibility criteria required participants to be at least 18 years old and proficient in either German or English. Participation was entirely voluntary and no financial incentives were provided to minimize potential self-selection or bias based on extrinsic motivation. Written informed consent was obtained from all individuals prior to participation. The study was approved by the Ethics Committee of Charité—Universitätsmedizin Berlin (EA4/015/25) and was prospectively preregistered with the German Clinical Trials Register (DRKS; https://drks.de/search/en/trial/DRKS00035946) in February 2025. Recruitment was based on non-probability convenience sampling, with efforts made to cast a wide net across various contexts throughout Germany. Distribution channels combined community-based outreach and online dissemination, including addiction support services, NGOs working in prevention, harm reduction, and (sexual) health services, peer networks, social media (e.g., *Instagram*, *Reddit*), newsletters, and mailing lists associated with clubs, festivals, party collectives, and magazines (see Supplementary Table S1 for an overview of survey distribution pathways).

### Assessments

The survey comprised validated instruments and study-specific items that were developed in response to gaps in existing measures. Where possible, the item development was informed by prior research on other substances (e.g., GHB/GBL [30, 31]). To ensure conceptual clarity, applicability, and practical relevance, the survey was designed in close collaboration with experts from harm reduction, prevention, addiction care, emergency care, psychotherapy, psychiatry, nightlife, and community stakeholders.

Sociodemographic characteristics were assessed using an established item set (e.g., [31]). Current mental disorders (present / not present) were specified with an open-text field. Depressive symptoms (PHQ-2 subscale) and anxiety symptoms (GAD-2 subscale) were screened using the four-item *Patient Health Questionnaire-4* (PHQ-4; [32, 33]), rated on a four-point Likert scale. Subjective mental and physical health were rated via visual analog scales (0–100), adapted from Balestroni and Bertolotti [34].

The survey employed parallelized item sets distinguishing between “synthetic cathinones that are usually smoked” and “synthetic cathinones that are usually snorted / consumed nasally”, each accompanied by consistent substance examples to reinforce this distinction throughout the survey. For reasons of feasibility, substances were clustered according to the most typical route of administration based on interdisciplinary expertise. Accordingly, the category “synthetic cathinones that are usually smoked” was intended to reflect common patterns of use and substance profiles (e.g., smokable characteristics of pyrovalerone-type cathinones), but it cannot be ensured that all participants who responded to items referring to this category had personally consumed these substances via smoking. For distinct substances that are particularly known in Germany (3-MMC, 4-MMC, and Monkey Dust), routes of administration were additionally assessed in greater detail; up to three additional open-text fields allowed participants also to specify further substances and their routes of administration using the same response options.

Frequency of use for usually smoked and usually nasally consumed synthetic cathinones, as well as other substances, was measured using seven-point ordinal scales (ranging from *“never”* to *“(almost) daily”*), previously employed in studies on other substances (e.g., [30, 31]). Details on use patterns and contexts were collected only from participants who reported use of smokable synthetic cathinones within the past 12 months.

Use settings were assessed via six Likert-scale items [30, 31]. Consumption motives were measured using 18 items adapted from the validated questionnaire by Boys et al. [35, 36]. Screening for problematic or dependent use included the four-item *CAGE*—*Adapted to Include Drugs* (CAGE-AID [37]) and a six-item checklist of ICD-10 dependence criteria (adapted from BZgA [38]), both assessed via binary (yes/no) responses. Specific aspects of use patterns (e.g., average dosage, session duration, redosing, binge-use, safer use techniques) were captured using a combination of quantitative and open-text responses. Concomitant substance use, including specific substance combinations and reasons for their joint use, was assessed using a binary item (yes/no; indicating whether respondents typically combine substances) and open-text items for elaboration. Adverse effects and other complications were assessed with binary items and open-text descriptions. Self-reported reasons and steps taken to reduce or discontinue use as well as perceived support needs and experiences with support services were explored using binary items and corresponding open-text elaborations, adapted from prior research on GHB/GBL [30].

### Analyses

All statistical analyses except for sensitivity power analyses were conducted using *IBM SPSS Statistics Version 29* [39]*.* The analyses primarily followed a descriptive approach to characterize sociodemographic and health-related variables, patterns of use, settings, motives, adverse effects, and support needs related to the use of smokable synthetic cathinones. Participants who had reported use within the past 12 months (*N* = 107) were grouped into *occasional use* (less than once per week) *and frequent use* (one or more times per week) to facilitate interpretable comparisons based on use frequency as use frequency is an established indicator of use severity and is associated with differences in risk profiles and psychosocial correlates [40, 41]. A weekly threshold was chosen to approximately distinguish between more infrequent situational vs. habitual high-intensity use patterns, in line with studies on other substances, including cannabis [41] and GHB/GBL [30, 31].

Inferential statistics were applied where appropriate and where the sample size was sufficient (considering overall attrition across the survey). Missing data were handled using pairwise deletion. Given the exploratory focus of the study, the limited sample size, and variation in the number of valid responses across items (as no forced responses were implemented in the survey to reduce the risk of insincere answers and dropouts), this approach was chosen to maximize the use of available data without introducing additional assumptions required for imputation approaches. Accordingly, the number of valid responses is reported for each item in the text, tables, and figures. A two-tailed significance level of α = 0.05 was applied to all inferential tests, with *p*-values adjusted for multiple testing using Holm’s sequential Bonferroni procedure within families of related analyses. Additionally, sensitivity power analyses were conducted in R (version 4.5.2) using the *pwr* package [42] to estimate the minimum effect sizes detectable given the available sample sizes in the present study, with 80% power at a two-tailed α = 0.05. Differences in use frequency by gender and sexual orientation were examined using chi-square tests (with Monte Carlo simulation in case of small expected cell counts). Spearman’s rank correlations were used to assess associations between use frequency and age and the frequency of use of other substances. Group differences in settings, motives, and indicators of problematic or dependent use were examined descriptively via cross-tabulations.

Open-ended responses were analyzed using inductive content analysis. Given the exploratory nature of the data and the relatively small number of responses per item, categories were developed inductively to identify recurring patterns related to use characteristics, motives, adverse effects, reduction efforts, and support needs. The resulting category structure and interpretations were discussed with an independent rater to enhance interpretative rigor. Category frequencies are presented descriptively, accompanied by illustrative examples.

## Results

### Use frequency and sociodemographic characteristics

#### Use frequency

A total of 1,548 individuals participated in the survey and answered at least the frequency of synthetic cathinone use. Of these, 88 individuals (5.7%) reported having used smokable synthetic cathinones more than 12 months ago. Among those with current or recent use, 52 (3.4%) used less than once per month, 28 (1.8%) 1–3 times per month, 18 (1.2%) 1–2 times per week, 4 (0.3%) 3–5 times per week, and 5 (0.3%) reported (almost) daily use.^2^ Detailed information on use patterns, motives, and perceived risks was collected from all participants who reported use of usually smoked synthetic cathinones within the past 12 months (*N* = 107). This sample served as the basis for all subsequent analyses; however, the effective sample size varies across analyses due to item nonresponse and attrition. For further analysis, these participants were grouped into *occasional use* (“ < 1 × per month” and “1–3 × per month”; *n* = 80), and *frequent use* (“1–2 × per week”, “3–5 × per week,” and “(almost) daily”; *n* = 27).

#### Sociodemographics

Table 1 presents the sociodemographic characteristics of participants who reported use of usually smoked synthetic cathinones within the past year (N = 107). Ages ranged from 18 to 57 years (*M* = 34.48), with a higher density of participants in the late twenties to late thirties. The majority (71.0%) identified as male, 18.7% as female, and 7.5% as diverse or non-binary. The largest share of participants (45.8%) identified as homosexual, 27.1% as heterosexual, 15.9% as bisexual, and 8.4% reported another sexual orientation. The sample showed diverse employment and educational backgrounds, with a predominance of participants who were employed and held a university degree. The majority (68.2%) of participants lived in Berlin, which aligns with recruitment patterns, as most participants accessed the survey link via Berlin-based networks such as the Berghain community subreddit (*n* = 29, 31.0%), the Clubcommission (*n* = 28, 26.2%), and the harm reduction project sidekicks.berlin (*n* = 19, 17.8%); for a detailed overview of the recruitment channels see Supplementary Figure S2. Despite efforts to include pathways targeting individuals from the open drug scene and other use contexts (e.g., low-threshold outreach-based services), no participants were recruited through these channels.

**Table 1:** Sociodemographic characteristics of the final sample of individuals who reported use of smokable synthetic cathinones within the past 12 months (*N* = 107)

Sociodemographic variable	*n*	%
*Age* *(M* = *34.48, SD* = *9.16;* Range = 18–57; median = 33)	-	-
*Gender identity*		
Male	76	71.0
Female	20	18.7
Diverse or non-binary	8	7.5
Not reported	3	2.8
*Sexual orientation*		
Homosexual	49	45.8
Heterosexual	29	27.1
Bisexual	17	15.9
Other sexual orientation (e.g., pansexual or queer)	9	8.4
Not reported	3	2.8
*Relationship status*		
Single	52	48.6
Committed relationship	48	44.9
Other relationship status (e.g., casual/dating, complicated, polyamorous)	4	3.7
Not reported	3	2.8
*Employment status*		
Employed	63	58.9
Self-employed	13	12.1
Enrolled at university	13	12.1
Unemployed/work-seeking	10	9.3
In vocational training	4	3.7
Not reported	4	3.7
*Educational status (highest level of education)*		
University degree	70	65.4
High school diploma	16	15.0
Completed vocational training	8	7.5
Upper secondary certificate	3	2.8
Lower secondary certificate	5	4.7
No degree (yet)	2	1.9
Not reported	3	2.8
*State of residence*		
Berlin	73	68.2
Bavaria	4	3.7
Baden-Württemberg	3	2.8
Brandenburg	2	1.9
Hamburg	2	1.9
Hesse	2	1.9
Lower Saxony	1	0.9
Not reported	9	8.4

#### Mental and physical health

A third (13 of 39 respondents of this item) reported a current mental disorder (point prevalence), including depressive disorders (*n* = 6), anxiety disorders (*n* = 5), (complex) PTSD (*n* = 3), ADHD (*n* = 3), schizophrenia (*n* = 1), and poly-substance addiction (*n* = 1). In addition, about one quarter (10 of 39 respondents) scored above the GAD-2 cutoff, indicating clinically relevant anxiety symptoms, and 11 of 39 respondents exceeded the PHQ-2 cutoff, suggesting relevant depressive symptoms. Self-rated current mental and physical health were assessed using visual analogue scales ranging from 0 (worst imaginable state) to 100 (best imaginable state). On average, participants rated their mental health at 64.95 (*SD* = 20.19; range = 13–97; median = 68.00; *n* = 39) and their physical health at 70.00 (*SD* = 20.86; range = 5–100; median = 74.00; *n* = 39).

#### Associations between use frequency and sociodemographics

**Fig. 1:**
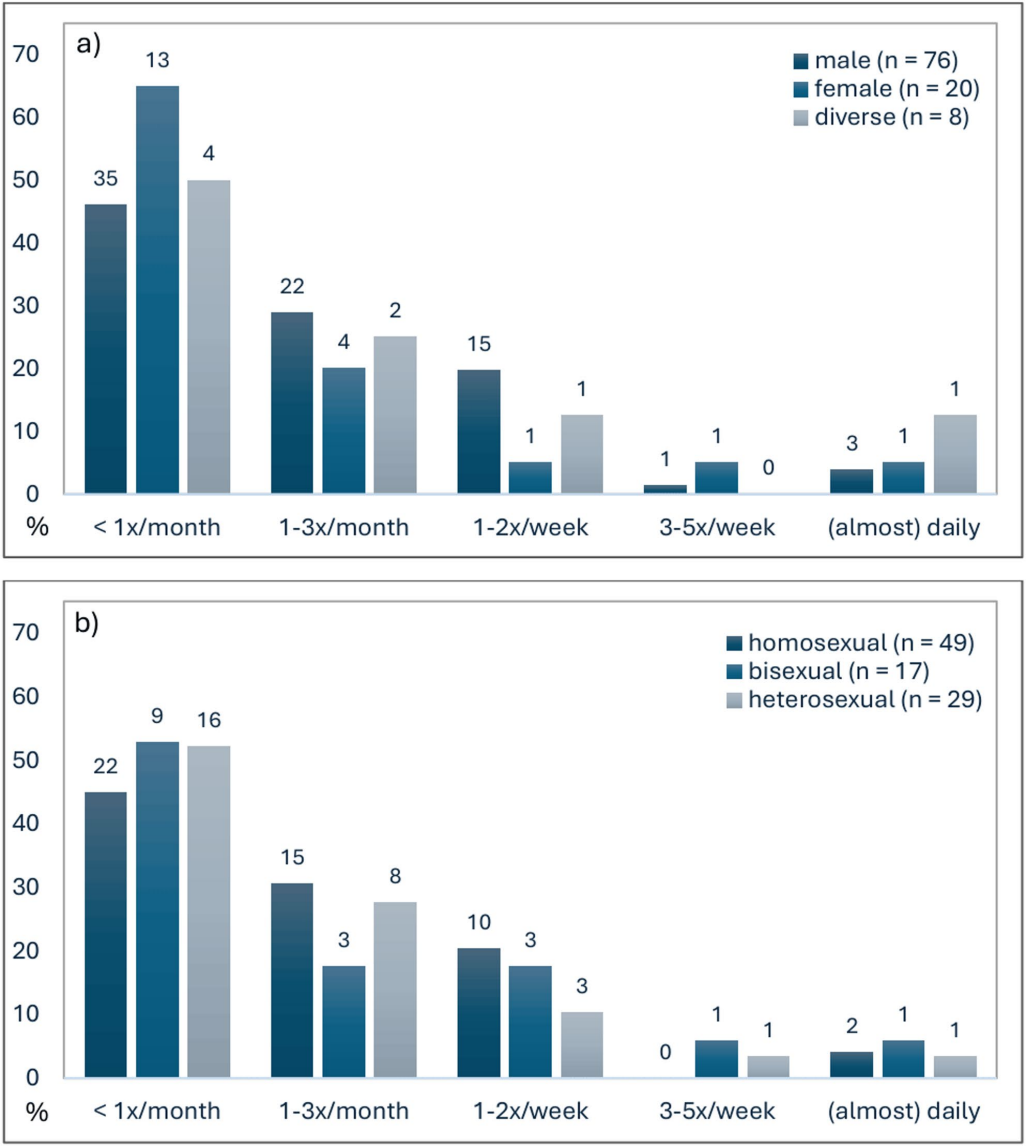
Reported use frequencies of smokable synthetic cathinones presented separately by gender identity (**a**) and sexual orientation (**b**). *Note*. Numbers above the bars indicate absolute frequencies within each gender or sexual identity group, whereas bar height reflects the relative proportion within each group

Descriptive crosstabulations for use frequency by gender identity indicated that men and non-binary participants tended to report slightly higher use compared to women (see Fig. 1; *n* = 104). However, observed differences were minor, and some category group sizes were very small. Due to low expected cell counts violating the assumptions of the chi-square test, significance was estimated using a Monte Carlo simulation (10,000 samples; *p* = 0.670, 99%-CI [0.558, 0.682], *p*_*Holm-adjusted*_ = 1.000; Cramer’s V = 0.094), which indicated no significant association. A similar pattern emerged for sexual orientation: although homo- and bisexual individuals were slightly more represented in the frequent use group, the chi-square test yielded no significant result, χ^2^(2, *n* = 95) = 0.993, *p* = 0.609, *p*_*Holm-adjusted*_ = 1.000; Cramer’s V = 0.102). Further, there was no significant correlation between age and use frequency, Spearman’s rho = -0.15, *p* = 0.166, *p*_*Holm-adjusted*_ = 1.000, *n* = 85. Sensitivity analyses indicated that, given the available sample sizes, the study had 80% power (two-tailed α = 0.05) to detect moderate to large associations, corresponding to correlations of approximately |ρ|≥ 0.28–0.30 and Cramer’s V ≥ 0.30. Smaller effects may therefore have gone undetected.

### Use settings

**Fig. 2:**
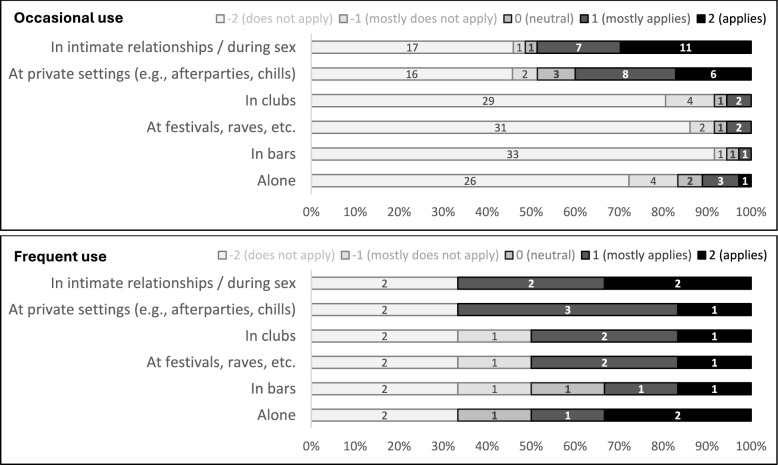
Distribution of settings of smokable synthetic cathinone use, grouped by use frequency. *Note*. Self-reported use frequency was categorized as occasional (“ < 1 × per month” and “1–3 × per month”; *n* = 36) vs. frequent use (“1–2 × per week”, “3–5 × per week”, and “(almost) daily”; *n* = 6). Numbers displayed within the bars indicate the number of respondents who selected the respective response option. Bar width represents the relative proportion within each use-frequency group. Given the very small size of the frequent-use group, findings are presented for descriptive purposes only. Categories may overlap, as afterparties and chills in chemsex contexts are commonly interpreted as “sex parties” (resulting in an overlap between “private settings” and “during sex”), and some club settings may also involve intimacy, sexual activity, or links to private afterparties [27, 43]

The most relevant contexts of occasional (*n* = 36) and frequent use (*n* = 6) were intimate relationships, sexual activity, and private settings (e.g., afterparties, chills; see Fig. 2). Solitary use was more frequently endorsed in the frequent use group, with 3 of 6 respondents rating this context as (mostly) applicable, compared with 4 of 36 respondents in the occasional use group. Club, festival, and rave settings were only rarely reported as relevant in occasional use, whereas half of the frequent-use group (3 of 6 respondents) indicated these contexts as (mostly) applicable. Across these reported patterns, use contexts may overlap, as some settings may co-occur or transition into one another.

### Motives

**Fig. 3:**
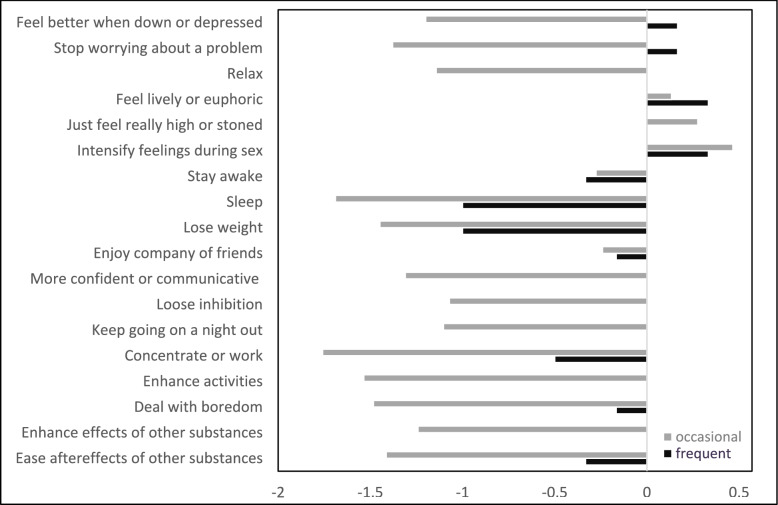
Arithmetic means of the ratings of reasons for smokable synthetic cathinone use, grouped by use frequency. *Note*. Self-reported use frequency was categorized as occasional (“ < 1 × per month” and “1–3 × per month”; *n* = 36) vs. frequent use (“1–2 × per week”, “3–5 × per week”, and “(almost) daily”; *n* = 6). Ratings for the extent to which each reason applied to the respondent ranged from -2 (“not at all”) to 2 (“completely”); for illustrative purposes, arithmetic means were calculated for each group while recognizing the ordinal scale of the ratings. If no bar is visible, the mean is 0. Given the very small size of the frequent-use group, findings are presented for descriptive purposes only

Respondents with occasional (*n* = 36) and frequent use (*n* = 6) most strongly endorsed using smokable synthetic cathinones to intensify feelings during sex, followed by achieving a lively or euphoric state (see Fig. 3). Those with occasional use additionally reported *“just feeling really high or stoned”* as a relevant motive. In contrast, those who reported frequent use also endorsed coping-related motives such as *“feeling better when down or depressed”* and *“stop worrying about a problem”*, which were rated as highly inapplicable by the occasional use group. Other motives (e.g., reducing inhibitions, relaxing, enhancing the effects of other substances) were generally rated as inapplicable among occasional use and more neutrally among frequent use.

The qualitative analysis of 22 open-text responses on the main reasons for using smokable synthetic cathinones identified sexual enhancement (e.g., *“get super horny”*, *“sex and we want to make it more… animalistic?”*) as the most frequently mentioned motive by 11 respondents. Euphoria and availability were each cited four times, while psychological self-medication (e.g., *“started off with sexdates, but soon became a desire to smoke as a way to cope with anxiety and depression”; “self medicating ADHD”*), peer pressure, and curiosity were each mentioned twice. Mislabeling *(“cause they sell [it] as t [i.e., “tina”/ methamphetamine]”*) and carelessness were each mentioned once as reasons, as well as personal ambivalence (*“I never want it, but then I stumble over myself because I’m somehow into it”).*

### Use patterns

#### Routes of administration

Among the 35 respondents who answered the route of administration for Monkey Dust, 31 reported smoking/vaping (two of whom additionally reported nasal use and one reported rectal use). The remaining four participants reported nasal use of Monkey Dust only. One additional participant who did not report Monkey Dust use indicated smoking/vaping for the smokable synthetic cathinone NEP in an open text field, resulting in a total of 32 out of 36 respondents reporting smoking/vaping of at least one smokable synthetic cathinone. It remains unclear whether (some of) the remaining four respondents also smoked synthetic cathinones but did not report this, or did not smoke them.

#### Dosage

The amount of smokable synthetic cathinones typically consumed per session ranged from 0 to 2000 mg, with a mean of 488.07 mg (*SD* = 674.65; median = 250.00; *n* = 15). The duration of typical use sessions ranged from 0 to 72 h, with a mean of 20.57 (*SD* = 16.51; median = 18.00; *n* = 23).

#### Redosing

A third (11 of 33 respondents) reported a strong urge to take more when using smokable synthetic cathinones (*“Strongest with Monkey Dust. Sometimes it starts as soon as ten minutes after use”; MDPHP is my daily use substance. I get the urge to redose when I'm more idle. Less so when I'm busy with something”*). Additionally, 4 respondents reported a moderate urge, 4 a mild urge, and 14 no urge to redose. Regarding loss of control, 5 of the 33 respondents reported always using more than intended during a typical session, 6 reported doing so often, 10 sometimes, 3 rarely, and 9 never.

#### Problematic or dependent use

**Fig. 4:**
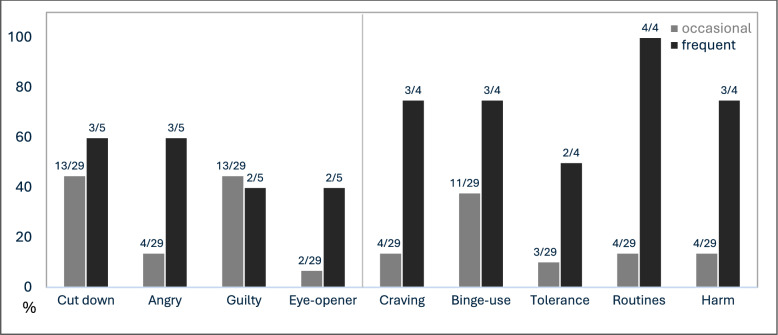
Positive responses to screening items on problematic/dependent use of smokable synthetic cathinones by use group. *Note*. Self-reported use frequency was categorized as occasional (“ < 1 × per month” and “1–3 × per month”) vs. frequent use (“1–2 × per week”, “3–5 × per week”, and “(almost) daily”). Numbers above the bars indicate positive responses per total item responses. Given the very small size of the frequent-use group, findings are presented for descriptive purposes only. The four CAGE-AID-items cover whether the respondents had ever felt they should *cut down* their use, gotten *angry* because someone criticized their use, felt *guilty* about their use, or used in the morning as an *eye-opener* to steady their nerves or get rid of a hangover. Further, five items adapted from the BZgA ICD-10 checklist assess core indicators of problematic or dependent use, including *craving*, *binge-use* / loss of control, *tolerance* development, structuring daily *routines* to enable substance use, and continued use despite physical, psychological, or social *harm* (the sixth checklist-item overlaps with the CAGE-AID “cut down” item and is therefore omitted)

A substantially higher proportion of respondents with frequent use endorsed screening items indicative of problematic or dependent use compared to those with occasional use (see Fig. 4). This was especially pronounced for items such as anger in response to criticism of their use, using as an eye-opener, craving, binge-use, tolerance development, making plans to accommodate substance use, and continued use despite harm. Those with occasional use most frequently endorsed the desire to cut down, feelings of guilt (at rates similar to the frequent use group), and binge-use, while all other indicators were reported less often. Three of the 5 respondents with frequent use (60.0%) and 4 of 29 with occasional use (37.9%) met the CAGE-AID cutoff (≥ 2), indicating likely problematic use [37]. According to the ICD-10 screening criteria, 3 of 4 respondents with frequent use (75.0%) and 4 of 29 with occasional use (13.8%) met the diagnostic screening threshold (≥ 3), indicating likely substance dependence [38].

#### Use of other psychoactive substances

The frequency of usually smoked synthetic cathinone use within the past year was significantly positively correlated with the use of usually nasally consumed synthetic cathinones (e.g., “mephedrone”/4-/3-/2-MMC; see Table 2; *n* = 99). The second strongest positive association was observed with amphetamine use; however, this association did not remain statistically significant after adjustment for multiple testing. Small positive but non-significant correlations were also observed with use of benzodiazepines, crack cocaine, methamphetamine, and GHB/GBL. In contrast, small negative (non-significant) associations emerged with cannabis and alcohol use.

**Table 2:** Non-parametric correlations between self-reported frequency of smokable synthetic cathinone use and frequency of use of other substances (*n* = 99)

Substance (correlated with smokable synthetic cathinone use)	Spearman’s rho	*p*-value
Typically snorted synthetic cathinones (e.g., 4-MMC, 3-MMC, etc.)	0.330	< .001**
Amphetamine	0.229	0.021
Benzodiazepines	0.197	0.052
Crack cocaine	0.172	0.091
Methamphetamine	0.169	0.099
GHB/GBL/BD	0.163	0.108
Potency drugs (not medically prescribed)	0.114	0.261
Other research chemicals (4-Fa, MXE, 5-MeOMiPT, “tusi”, etc.)	0.101	0.325
Synthetic Cannabinoids	0.085	0.415
LSD	0.083	0.412
Opioids	0.079	0.440
Z-drugs	0.056	0.586
Anabolic steroids (not medically prescribed)	0.038	0.712
2C-B	0.012	0.910
Psilocybin	-0.024	0.815
MDMA	-0.039	0.694
Cocaine	-0.066	0.514
Ketamine	-0.072	0.469
Cannabis	-0.121	0.234
Alcohol	-0.143	0.224

Substance use frequency was assessed on a 7-point ordinal scale ranging from “never” to “(almost) daily” (with smokable synthetic cathinone use restricted to use at least within the past year due to inclusion criteria). The table presents uncorrected *p*-values, while the significance levels indicated by asterisks refer to Holm-adjusted *p*-values; significance levels: ***p < .001, **p < .01, *p < .05

#### Concomitant substance use

About half of respondents (19 of 35 responses) reported combining smokable synthetic cathinones typically with other substances. GHB/GBL was most frequently co-used (reported by 13 of the 17 individuals who specified the co-used substances), followed by 6 citing 4-/3-MMC, 3 co-using methamphetamine, and 2 (crack) cocaine. The analysis of 13 open-text responses on reasons for concomitant use revealed three main motives: 1) to *enhance sexual pleasure* (cited by 4 respondents as reasons for combined use with substances such as GHB/GBL, 4-/3-MMC, methamphetamine, and cocaine); 2) to *intensify the high* (reported by 3; e.g., regarding GHB/GBL, cocaine, 4-/3-MMC, poppers); and 3) to *attenuate effects* (named by 3 respondents; e.g., regarding GHB/GBL, 4-/3-MMC, alcohol, benzodiazepines; e.g., *“GHB to counteract the tweaky side effects”* or *“to calm down a bit from the high of monkey dust”*).

#### Safer use

Eleven of 31 respondents reported practicing safer-use strategies when using smokable synthetic cathinones (e.g., *“hygiene”*, *“using personal utensils”*, *“measuring doses”*, *“buying from very well-trusted people”*).

#### Initiation

The age of first use of smokable synthetic cathinones ranged from 19 to 52 years (median = 31.00; *M* = 33.74, *SD* = 9.19; *n* = 21). The most commonly reported context for first contact was within sexual settings (reported by 9 of 12 respondents). These could be further differentiated into chemsex-typical group sex and sex party events (*n* = 7; e.g., *“sex party”*, *“at a chill”*, *“at an orgy”*) and *“hook-up dates”* (*n* = 2). Additional initiation contexts included party environments (*n* = 2, *“raves”*, *“festival”*) and acquisition via dealers (*n* = 1; *“I was at my dealer’s place to buy other substances when it was offered to me”*).

### Adverse effects

One third (10 of the 32 respondents) reported negative consequences under the influence or as a result of smokable synthetic cathinone use. The proportion was higher among frequent use (2 of 4 respondents) than occasional use (8 of 28). Four of the 10 individuals who provided open-text responses reported paranoia, 3 individuals panic or anxiety attacks, and 2 an uncomfortable social setting; further adverse experiences (each cited once) included prolonged sensory disturbances lasting for weeks, severe weight loss, detachment from reality, sexual encounters with individuals whom the person disliked, and sexualized violence.

### Motives and strategies of reduction or cessation

#### Motives

Half of the respondents (16 of 32) reported having reasons to reduce or cease their use of smokable synthetic cathinones. In 12 open-text responses, the most frequently mentioned motives were concerns about their own physical and mental health, deterrence through observing others (*“I have seen friends become shadows of their former selves in a matter of months”, “I have seen the psychological and addicting effects on acquaintances”*), and to avoid loss of control. Further reasons included experiences of paranoia, depersonalization, and risky or boundary-violating behavior, both self-experienced or witnessed (*“Paranoia. The feeling of not recognizing myself anymore. Increasingly risky behavior. Unpredictable behavior in co-users”, “Loss of reality, loss of control, sexualized violence”*).

#### Strategies

Twelve of the 32 respondents of this item had already taken steps to reduce or discontinue their use of smokable synthetic cathinones. The most commonly mentioned strategies included avoiding consumption contexts (reported by 4 of 11 respondents; e.g., *“I've tried to eliminate dating apps from my phone and certain contacts that lead me down that road”*, *“preparing to leave Berlin to get away from situations they are used in”*), complete abstinence (*n* = 3), psychotherapy (*n* = 3), attending self-help groups such as *Narcotics Anonymous* (*n* = 1), self-education about the negative effects (*n* = 1), and following self-imposed rules regarding duration, quantity, and frequency of use (*n* = 1).

#### Support services

Five of the 32 respondents reported a need for support regarding their use of usually smoked synthetic cathinones (e.g., *“more awareness”*, *“public campaigns”*, *“self-help groups”*). These same respondents already accessed support services, including therapy, queer counseling services, and self-help groups (e.g., *Crystal Meth Anonymous* and *Narcotics Anonymous*).

## Discussion

### Key findings and interpretations

This study constitutes the first detailed, user-centered investigation of sociodemographic characteristics, use patterns, underlying motives, perceived harms, and support needs associated with the use of smokable synthetic cathinones, employing a primarily descriptive-exploratory design that integrates quantitative and qualitative elements.

#### Use patterns

Approximately one eighth of individuals who reported lifetime use of usually nasally consumed synthetic cathinones (e.g., mephedrone, 4-/3-/2-MMC, 4-/3-CMC) also reported having used usually smoked synthetic cathinones (e.g., Monkey Dust). This suggests that smokable synthetic cathinones represent a comparatively niche subclass of substances, even within populations already engaged in cathinone use. Combined with their disproportionately high representation in clinical and emergency care settings, this highlights the need for targeted research and tailored intervention strategies focusing specifically on this subclass.

The sample of 107 individuals who reported use of smokable synthetic cathinones within the past 12 months exhibited a broad spectrum of use frequencies, ranging from single or irregular situational use to (almost) daily use—suggesting distinct use profiles. While anecdotal accounts and case studies from clinical and harm reduction contexts often emphasize a predominance of frequent problematic use [1, 4, 15, 25], such patterns appeared less prominent within the present sample, with only about one quarter of respondents reporting frequent use (defined as at least weekly use). Also regarding redosing urge and loss of control, the pattern within the sample appeared more heterogeneous than portrayed in anecdotal accounts [12] and than expected based on pharmacological characteristics [19]: while approximately one third reported a very strong urge to redose and frequent loss of control, around half described only mild or no urge to redose and one third reported little to no loss of control during typical use sessions. This suggests that the compulsive high-risk use patterns often associated with smokable synthetic cathinones may not be universally applicable and likely also depend on individual and contextual factors. Nonetheless, it is important to consider potential risks early on and across all use groups, as harm is not limited to frequent use [1]—a point further underscored by the finding that one third of respondents reported adverse consequences, despite only one quarter indicated frequent use.

#### Sociodemographic characteristics, health-related aspects, and use settings

The majority of participants who used smokable synthetic cathinones shared key characteristics commonly described in previous research on chemsex, including male gender, homosexual or bisexual orientation, and high educational status [43]. Reported consumption settings and motives further support the classification of chemsex as a central context of smokable synthetic cathinone use. So-called "chemsex-chills", party-settings, and intimate relationships were most frequently named as contexts of use, and sexual motives were rated as the most relevant reason for consumption. Patterns of concurrent substance use—particularly the frequent use of GHB/GBL, nasally consumed synthetic cathinones, methamphetamine, amphetamine, and (crack) cocaine—also align with chemsex profiles [27, 43]. Sex-related reasons were most commonly given to explain concomitant polydrug use, and the average duration of use sessions was 20 h, which corresponds with existing knowledge from the scene, where chemsex sessions are known to last multiple hours or even days [27, 43].

Despite the dominance of a chemsex-typical profile in the sample, a notable share of participants did not fit this pattern. Specifically, about one fifth identified as female and one quarter as heterosexual—and were also represented among those with frequent use, albeit slightly less often (with differences in use frequency by gender and sexual orientation not being statistically significant). These findings align with informal accounts of smokable synthetic cathinone use outside of sexual contexts, such as the so-called “Flex” scene in Göttingen [2], which (e.g., unlike the Berlin chemsex context) is linked to homelessness and social precarity (notably, this group was not reached in the present study, despite efforts to disseminate the survey nationwide in low-threshold support settings, including Göttingen). These findings of heterogenous profiles underscore the need to broaden the scope of education, prevention, harm reduction, and treatment efforts. While targeted interventions for chemsex use remain important, they should be complemented by strategies tailored to other use profiles and settings – particularly those involving social marginalization, structural vulnerability, use in public/open drug scenes, or non-urban areas. Such efforts will be crucial to adequately respond to the heterogeneous realities of smokable synthetic cathinone use and to prevent the neglect of use groups who fall outside the typical chemsex contexts [44].

The mental and physical health indicators observed in the sample possibly hint to a heightened level of vulnerability among individuals using smokable synthetic cathinones. One third reported a current mental disorder and over one quarter screened positive for clinically relevant symptoms of depression or anxiety. These rates surpass those typically found in representative surveys of the general population in Germany [45]. While self-rated mental and physical health status averaged in the mid-to-upper range, the wide range of responses indicates notable heterogeneity. In summary, these findings highlight the relevance of integrated interventions that consider both substance use and (mental) health concerns.

#### Motives

Participants endorsed a range of motives; sexual enhancement and euphoria were the most frequently reported in both use groups, while those with frequent use additionally stated coping motives—such as mood regulation and distraction from problems. These findings align with established models of *dual-process motivation*, where *positive reinforcement* (euphoria, sexual confidence) and *negative reinforcement* (relief from negative affect) both drive substance use but differ in salience by use pattern [46, 47].

#### Adverse effects and counterstrategies

Compared to occasional use, those reporting frequent use markedly more frequently met criteria for problematic or dependent use: over six times as many exceeded the ICD-10 threshold, and nearly twice as many met the CAGE-AID cutoff. Only one participant reported a diagnosis of substance dependence, contrasting the high rates of screening-positive cases—especially among frequent use. This gap may reflect underdiagnosis, limited treatment access, or low self-recognition, as seen in other (marginalized) substance use contexts [48].

A variety of harms was reported—most commonly paranoia, psychosis, anxiety attacks, and social disruptions—with a higher share among those who reported frequent use. These findings align with international case reports, (post-mortem) forensic data, and animal models linking pyrovalerones to acute psychosis and dissociative or aggressive behavior [8, 24, 49], and extend this evidence by providing user-level perspectives from a community sample. The results underscore the need for early risk detection and targeted support strategies that address the acute, mid- and long-term (psychological) effects of pyrovalerones, in- and outside clinical contexts. Special emphasis may be placed on pyrovalerone-related psychosis, as drug-induced psychoses are often particularly severe, with evidence suggesting an increased risk of developing psychotic disorders following such episodes [50], highlighting the importance of continued research and awareness in this area.

The main reasons respondents gave for wanting to reduce or discontinue use largely reflected these adverse effects. This further points to the necessity of expanding preventive, harm reduction, and treatment options tailored to the specific risks and experiences associated with this substance class. About one third of the respondents reported using safer use strategies when consuming smokable synthetic cathinones. While this indicates a degree of risk awareness, it also suggests that the majority do not engage in such harm reduction practices, which again underscores the need for targeted education and low-threshold interventions, especially for use populations that may currently be underserved.

### Strengths and limitations

This study is characterized by a broad recruitment strategy, interdisciplinary development, and an anonymous design that may have helped reduce socially desirable responding. As outlined in methodological discourse on the use of non-probability sampled, anonymous web-based surveys in drug research [16, 51] studies on hidden populations and stigmatized topics often rely on convenience-based approaches, prioritizing analytic utility and contextual insight over statistical representativeness. Against this background, several methodological limitations of the present study should be acknowledged.

The use of convenience sampling limits generalizability and may introduce selection bias. For example, individuals with higher digital literacy and more stable living conditions may have been more likely to participate, while people with lower socioeconomic status, high-risk substance use, and severe health-issues may be underrepresented. This limitation is further underscored by the lack of successful recruitment via low-threshold outreach-based services and other pathways targeting individuals from the open drug scene and related use contexts, suggesting that relevant—and potentially particularly vulnerable—populations were not adequately captured. Future studies should therefore aim to reduce participation barriers by employing additional outreach-based recruitment strategies, shorter assessments [51], offering non-digital participation options, and providing extrinsic incentives (e.g., financial compensation) to facilitate the inclusion of these populations.

Due to the cross-sectional and observational nature of the study, no causal inferences can be made, and unmeasured confounding variables may have influenced the findings. Skipped items and increasing item nonresponse over the course of the survey reduced the analyzable sample size, particularly for later sections, thereby limiting the statistical power and interpretability of (subgroup) findings. Accordingly, sensitivity analyses indicated that the available sample sizes were only sufficient to detect moderate to large effects, whereas observed associations were small. Attrition may be partly due to the questionnaire length, which could have disproportionately affected participants with reduced cognitive capacity. In addition, item nonresponse substantially constrained the scope and depth of the qualitative analyses intended to complement the quantitative findings. While the open-text data provided relevant insights, the small number of responses and overlaps between inductively derived categories limited systematic qualitative interpretation.

The reliance on self-reported data entails the usual risks of recall inaccuracies and response bias. In addition, particularly with synthetic cathinones, the rate of mislabeling and contamination is high [12], making it unclear from self-reported data (without toxicological information) whether individuals who report using pyrovalerones have indeed consumed pyrovalerones or possibly other psychoactive substances. Furthermore, the group of smokable synthetic cathinones itself is large and heterogeneous, encompassing substances with differing pharmacological effects and risk profiles [15, 21, 52, 53]. For reasons of feasibility, these substances were clustered in the survey, which may have introduced additional imprecision. In this context, it also cannot be ensured that all respondents who answered items on smokable synthetic cathinones actually smoked them, which is relevant given differences in effects and risk profiles across routes of administration [54, 55]. Future research may build on these insights and expand methodological efforts to further explore this underexamined topic.

## Conclusions

This study is the first to offer detailed, user-centered insights into the use of smokable synthetic cathinones, revealing a heterogeneous population with both chemsex-related and non-chemsex patterns. While many respondents reported infrequent and situational use, a notable subset exhibited signs of high-frequent and problematic/dependent patterns, alongside considerable psychological distress and adverse effects. These results underscore the need for differentiated prevention, harm reduction, and treatment strategies—tailored not only to chemsex use but also to other emerging use profiles and settings.

Beyond its empirical contributions, this study holds broader relevance for harm reduction services, clinical practice, and public health. As the landscape of synthetic psychoactive substances continues to evolve, integrating user-level perspectives becomes critical for designing effective, context-sensitive responses. Understanding underlying motives and how individuals perceive and manage risks may help shape more targeted interventions, especially in high-risk and underserved settings.

## Supplementary Information

Supplementary Material

## Data Availability

All data, analysis code, and materials are available upon reasonable request from the corresponding author; due to the sensitive nature of the data, full open sharing is not possible.

## Footnotes

1 *N-ethylpentedrone* (NEP) is a synthetic cathinone that can be effectively smoked [11]. While structurally distinct from pyrovalerones, NEP shares similar pharmacodynamic properties. Although detected in some drug checking samples in Berlin [12], NEP appears to be less prevalent than several pyrovalerones to date.

2 The 195 individuals who indicated lifetime use of usually smoked synthetic cathinones represent 13.3% of the 1,446 individuals with lifetime use of usually nasally consumed synthetic cathinones (e.g., 3-MMC, 4-MMC, etc.). Only one respondent with lifetime use of usually smoked synthetic cathinones reported never having used usually nasally consumed variants.

## Declarations

### Clinical trial registration

The study was pre-registered in the German Clinical Trials Register on 02/2025 (drks.de/search/en/trial/DRKS00035946).

### Generative AI and AI-assisted technologies in the writing process

Generative AI and AI-assisted technologies (e.g., ChatGPT) were used solely to improve the readability and language of the manuscript. No AI tools were employed to generate content, develop ideas, perform analysis, or contribute to the interpretation of findings. The authors take full responsibility for the content.
