# Supplementary material for: From dust till dawn: patterns, motives, and risks of using smokable synthetic cathinones
Source: Harm Reduct J. 2026 Feb 25;23:44. doi: 10.1186/s12954-026-01428-8 (PMC12961792; doi:10.1186/s12954-026-01428-8)
Supplement: Supplementary file 1 — Supplementary Material [file 12954_2026_1428_MOESM1_ESM.pdf]

Supplemental Material

From Dust till Dawn:
Patterns, Motives, and Risks of Using Smokable Synthetic Cathinones

Figure S1. Evolution of public interest in "Monkey Dust" based on Google Search queries.

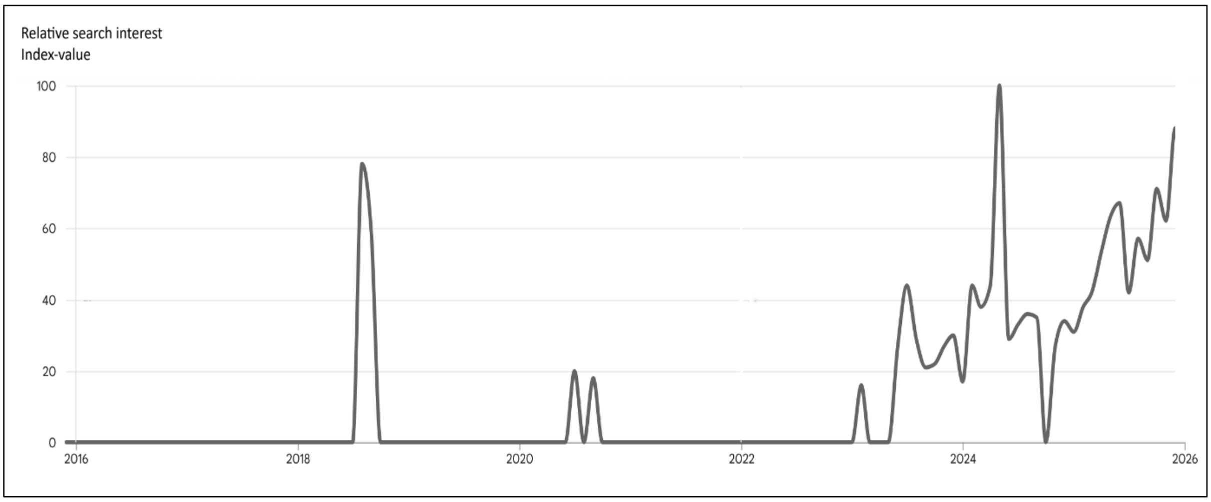

Note. Data were retrieved from Google Trends (https://trends.google.com/trends/) and reflect how often the term "Monkey Dust" was searched for on Google in Germany over the past ten years (1 January 2016 to 31 December 2025), with values normalized on a scale from 0 to 100 (an index-value of 100 indicates peak search frequency), reflecting relative rather than absolute search volumes (Google does not publicly provide absolute search counts). The first isolated peak in August 2018 is likely attributable to increased public attention rather than to a rise in use in Germany, as it coincides with sensationalized media coverage on "Monkey Dust" in Stoke-on-Trent, a city in the UK. From 2023 onwards, an upward trend can be observed. This pattern is consistent with converging anecdotal reports of increased pyrovalerone use in Germany across drug checking, addiction counseling services, emergency medical settings, toxicological analyses, and other sources. It provides further indication that the relevance of "Monkey Dust" in Germany has increased over the past three years (particularly in Berlin, where search activity is highest), while acknowledging that Google search activity does not necessarily reflect actual engagement in use (either through personal use or contact with individuals who use the substance).

Figure S2. Survey access routes of the final sample (*N* = 107).

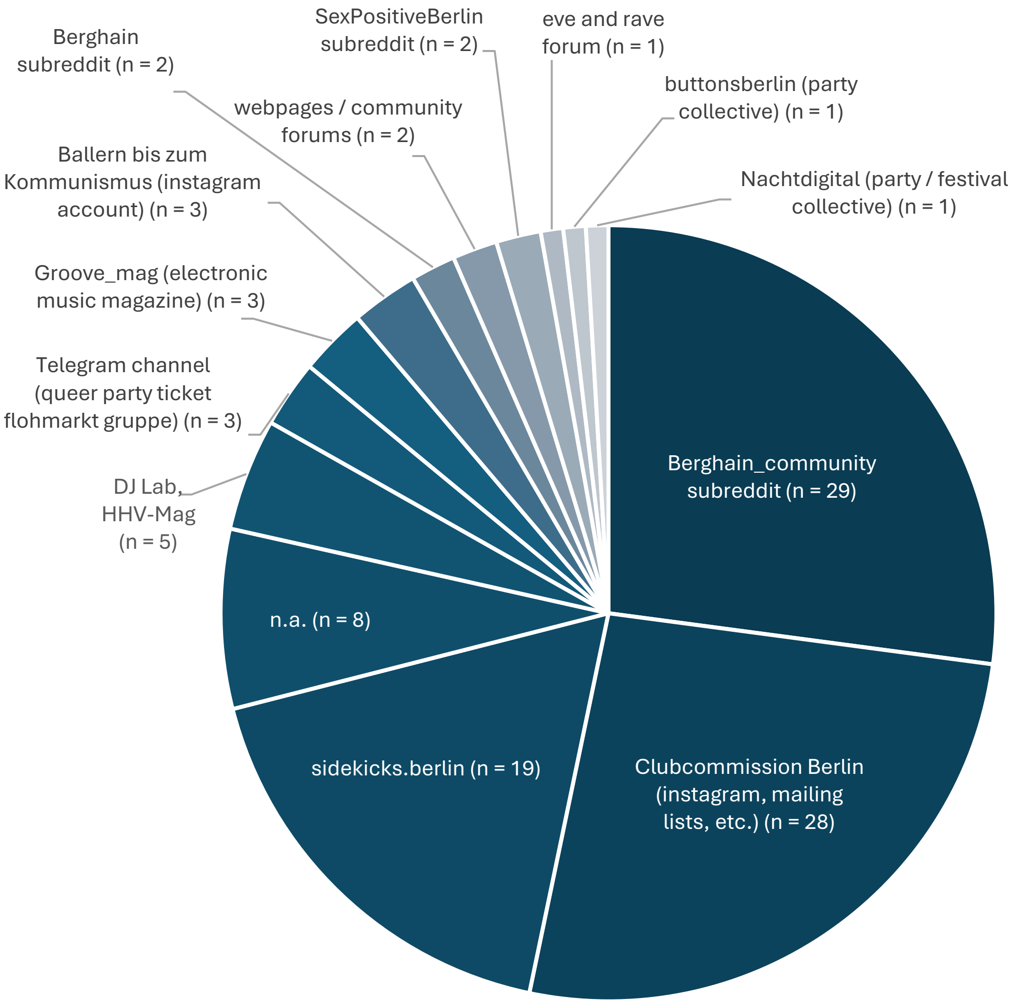

Note. Different link suffixes were used during recruitment, allowing the identification of access pathways.

Table S1. Overview of survey distribution pathways.

Drug counselling and addiction services, including harm reduction, community-based outreach, and low-threshold services

- Drug counselling services Berlin (Drogennotdienst Berlin; Fixpunkt Berlin; Vista Berlin)
- Drug counselling services Braunschweig (Drobs Braunschweig)
- Drug counselling services Bremen (Comeback Bremen)
- Drug counselling services Cottbus (Caritas Cottbus)
- Drug counselling services Dortmund (kick-dortmund)
- Drug counselling services Dresden (Drogenberatung Dresden)
- Drug counselling services Frankfurt (Drogennotdienst fam)
- Drug counselling services Gelsenkirchen (drogenberatung-kc)
- Drug counselling services Göttingen (Lukas-Werk-Gesundheitsdienste; Suchtberatung Göttingen)
- Drug counselling services Hamburg (Drop in Hamburg, DAH Hamburg)
- Drug counselling services Köln (Drogenhilfe Köln)
- Drug counselling services Leipzig (Scouts)
- Drug counselling services München (Suchtambulanz Caritas München)
- Drug counselling services Nürnberg (Mudra)
- Drug counselling services Rostock (Suchthilfe Rostock)
- Drug counselling services Stuttgart (Release)

Social media accounts, apps, forums, and services related to drugs

- Drogen subreddit
- knowdrugs app
- Drugchecking Berlin
- Eve and rave forum
- Other webpages / community forums

Chemsex / MSM ("men who have sex with men") scene

- Bundesinitiative Sexualisierter Substanzkonsum e.V. mailing-list
- Deutsche Aidshilfe / Facharbeitskreis Schwule Prävention IV
- ChemCon Congress 2025 in Berlin
- Lab.oratory subreddit
- Herrensauna.official (instagram account)
- Check Mag (health magazine for men)

Community-based support services focusing on sex work and associated substance use

- Sexworks Berlin
- BesD e. V. | Berufsverband Sexarbeit
- Smart Berlin / Mann-o-Meter
- Hydra Berlin

Social media accounts and mailing lists related to party, nightlife, clubs, festivals, and similar settings

- Festivals: Nachtdigital; Whole.festival; Fusion (internal mailing list)
- Social media accounts of clubs/collectives in Berlin: ButtonsBerlin; Multisex.me; about.blank; fandangoberlin; oxi.club; disko babel; Sisyphos; RSO; Club OST; Tresor
- Subreddits referring to Berlin clubs/nightlife: Berghain_community subreddit; Berghain subreddit; SexPositiveBerlin subreddit
- Clubs/collectives in Hamburg: Südpol HH (internal mailing list)
- Clubwear/-fashion: nakt_studio
- Telegram channels/groups: Berghain Telegram group; queer party ticket flohmarkt gruppe
- Ballern bis zum Kommunismus (instagram account posting memes on substance use and partying)
- Online magazines and music blogs: groove_mag; DJ Lab, HVV-Mag

Awareness and other safer party initiatives

- SONICS Safer Nightlife Dachverband
- Basis Frankfurt

Note. Recruitment was primarily conducted via social media (e.g., posts on Instagram, Reddit), while additional pathways included posters/flyers in service settings, mailing lists, websites, online magazines, and other channels. Pathways highlighted in blue indicate channels through which participants of the final sample accessed the survey.
